# Early intervention in eating disorders: introducing the chronopathogram

**DOI:** 10.1007/s40519-025-01715-6

**Published:** 2025-01-23

**Authors:** Antonio Preti, Francesco Bevione, Maria Carla Lacidogna, Andrea Raballo, Michele Poletti, Giovanni Abbate-Daga

**Affiliations:** 1 Eating Disorders Unit, Department of Neuroscience, University of Turin, Via Cherasco 15, 10126 Turin, Turin, Italy; 2 Chair of Psychiatry, Faculty of Biomedical Sciences, University of Southern Switzerland, Lugano, Switzerland; 3 Cantonal Sociopsychiatric Organisation, Mendrisio, Switzerland; 4 Department of Mental Health and Pathological Addiction, Child and Adolescent Neuropsychiatry Service, Azienda Unità Sanitaria Locale-IRCCS Di Reggio Emilia, Reggio Emilia, Italy

**Keywords:** Eating disorders, Anorexia nervosa, Bulimia nervosa, Early intervention, Neurodevelopment, Risk factors

## Abstract

Eating disorders (EDs) pose significant challenges to mental and physical health, particularly among adolescents and young adults, with the COVID-19 pandemic exacerbating risk factors. Despite advancements in psychosocial and pharmacological treatments, improvements remain limited. Early intervention in EDs, inspired by the model developed for psychosis, emphasizes the importance of timely identification and treatment initiation to improve prognosis. Challenges in identifying prodromal phases and measuring the duration of untreated illness highlight the complexity of early intervention efforts in EDs. Current research focuses on reducing the duration of untreated eating disorder (DUED) and understanding the cognitive and behavioral symptoms preceding ED onset. However, current early intervention programs for EDs showed mixed results, necessitating further investigation. We introduce here the chronopathogram, a tool that may aid in precisely investigating the role of development in EDs. A chronopathogram is a graphical representation of pathological events as they unfold over time. Understanding the neurodevelopmental aspects of EDs and utilizing tools like the chronopathogram can aid in tracking the unfolding of symptoms over time, facilitating early detection and intervention efforts. Overall, addressing the key factors influencing the onset and course of EDs is essential for effective early intervention in these conditions.

**Level of evidence**: Level V narrative review.

**Supplementary Information:**

The online version contains supplementary material available at 10.1007/s40519-025-01715-6.

## Introduction

Eating disorders (EDs) are characterized by severe disturbances in eating behavior, often accompanied by distorted perceptions of weight and body shape, which can result in various psychiatric and somatic complications [1]. The current nosography recognizes anorexia nervosa (AN), bulimia nervosa (BN), and binge eating disorder (BED) as the primary EDs [2, 3]. The co-occurrence of mental and somatic disorders in individuals with EDs significantly affects their quality of life and survival. People with EDs, particularly AN, experience increased disability and higher mortality rates [4, 5].

EDs are common among adolescents and young adults, with a higher incidence in females than males. Estimates suggest that 8.4% of women and 2.2% of men experience eating disorders (EDs) in their lifetime, with rates ranging from 3.3% to 18.6% for women and 0.8–6.5% for men [6]. In early adulthood, up to 18% of young women and up to 2.4% of young men may have experienced an ED [7]. Notably, the COVID-19 pandemic has been linked to an increase in the incidence of EDs [8, 9].

Despite evidence for the efficacy of some psychosocial and pharmacological treatments for EDs [10–12], improvements are often modest and time-limited [11, 13–15]. Currently, not more than 50% of those with an ED make a full recovery after treatment [1]. Thus, new avenues for research are advocated in support of new treatments [16, 17] and early intervention [18].

### Early intervention in eating disorders

Well before the advent of a dedicated early intervention model for psychosis (for a review [19]), professionals working on EDs had already championed the early identification and treatment of individuals at risk or suffering from such disorders [20–22]. The advocacy for early intervention in EDs grounded in some assumptions that became diffuse in mental health due to the early intervention model in psychosis: (a) a common occurrence of a time lag between illness onset and treatment admission; (b) an adverse prognostic impact associated with prolonged untreated illness; (c) the anticipation of intervention timing to enhance prognosis and treatment [23].

Earlier investigations underscored the detrimental outcome linked to a substantial delay between onset and initial treatment admission [23]. However, while the EDs domain lagged in producing studies focused on the initial episodes of AN or BN [24], confusion arose in outcome studies between the duration of untreated illness and the simple calculation of illness duration until case enrollment. A prolonged illness duration often reflects treatment non-response and insufficient illness insight, indicative of poor compliance with prescribed therapy. The duration of untreated illness represents the specific consequence of lacking treatment and the potentially toxic effects of the illness on the body, predisposing to a suboptimal treatment response when initiated [25]. The prognostic value of disease duration is unclear due to mixed data [26]. Duration of untreated illness in EDs shows an inverse relationship with the likelihood of remission, suggesting that shortening the time to access the service may improve clinical outcomes [27]. However, so far, evidence has been derived from retrospective studies. Retrospectively gauging the duration of untreated illness in EDs, particularly AN, proves challenging due to ego-syntonic symptom perception, patient concealment, and potential family collusion, known as “family accommodation”, until the disorder reaches a critical stage [28]. Barriers to help-seeking in EDs mirror those in psychosis, encompassing stigma, shame, denial, underestimation of illness severity, low motivation to change, and negative attitudes toward change and help-seeking itself [29, 30]. Moreover, initial positive effects linked to EDs—like AN as a mean of achieving a sense of egosyntonic psychological stability, security and control, the avoidance of negative emotions, and communication of feelings of distress to others—tend to make individuals less involved in the process of changing, fostering some degree of resistance to treatment [31–33]. Furthermore, during the weight loss phase, individuals often report an inner sense of strength, mastery, and skillfulness [32]. Additional hindrances include a lack of encouragement, unfamiliarity with available resources, and financial challenges in affording treatment. Conversely, emotional distress, evident mental health issues, and health concerns foster help-seeking behaviors [30]. Overall, in Europe, the median time between the onset of eating disorder symptoms and accessing a specialist unit for EDs is 2 years [34]. A longer delay, up to 5 years, has been reported in Australia [35]. The necessity for early intervention in eating disorders, thus, is grounded on strong clinical evidence.

Traditionally, early intervention programs for EDs focused on enhancing disorder recognition in general practice and relevant settings encountering patients with EDs [36, 37]. However, inspired by findings in psychosis [38, 39], the field of early intervention in EDs is rapidly advancing. One focus of the current research is the reduction of the duration of untreated eating disorder (DUED), proved as a modifiable factor related to outcome [40]. However, the definition and measurement of DUED and its components vary considerably between studies, and it is often difficult to establish the onset of the symptoms.

Clinical staging is even more complex [25]. There is evidence from longitudinal studies that some symptoms with onset in childhood, such as obsessive–compulsive, generalized anxiety, and social phobia, are associated with later development of an ED [41, 42]. Over-evaluation of weight/shape in one’s self-worth and feelings of fatness and fear of weight gain often precede the onset of an ED [43, 44]. These cognitive symptoms dominate the early stage of illness, while behavioral symptoms (restrictive dieting, compensatory conducts) tend to occur later [44, 45]. Nevertheless, it is undecided how much cognitive symptoms belong to the prodromal phase of the disorders or whether they are indicators of an already-onset illness. Moreover, so far, the theoretical conceptualization of a Clinical High risk for Eating Disorders (CHR–ED) has not translated into an etiological or therapeutic model [18].

Early intervention programs for EDs are rare [46]. The two most tested ones are the Maudsley Family Based Treatment (M-FBT), targeting patients with AN within the first 3 years of onset [47, 48], and the First Episode Rapid Early Intervention Service for Eating Disorders (FREED) program, designed for patients with an ED within the first 3 years of onset [49, 50]. Results are mixed. For the M-FBT, remission rates up to 80% have been claimed, but recovery rates are low at follow-up [51]. The FREED lacks a control group to corroborate the positive findings observed in the enrolled patients.

### Risk factors for EDs

Effective early intervention for eating disorders requires identifying predictors, particularly risk factors that may precede the onset of the full syndrome. Since the seminal article of Jacobi et al. [52], the investigation of risk factors for EDs has proceeded along two lines: the identification of psychosocial antecedents of the full or partial syndromes, and the investigation of a wide array of biological correlates, from supposedly fixed (genes and polymorphisms) to fluctuating until evanescence (gut microbiota) [53].

The role of inherited genetic risk in EDs seems supported by robust evidence from genome-wide association studies (GWAS) and twin studies [54–56]. However, sample sizes large enough to overcome the statistical limitations of genetic studies have been reached for AN only, and causal pathways between genes and biological mechanisms purportedly involved in EDs have yet to be elucidated [54, 55].

A history of abuse, trauma, and childhood obesity has been repeatedly related to EDs [53]. The most convincing evidence links childhood sexual abuse with BN and appearance-related teasing victimization with any eating disorder [14, 57, 58]. Nevertheless, many studies on the topic are cross-sectional or retrospective, and the direction of causality is far from being established, i.e., from trauma to the syndrome or from biological vulnerability to the trauma.

Comorbidities between EDs and other mental health disorders influence the severity of ED symptoms [53]. Therefore, comorbidity may serve as a staging marker, but its utility as a predictor of CHR–ED status requires confirmation through longitudinal studies, whose evidence is limited. Although anxiety disorders may predict an increased risk of AN, a longitudinal association between specific anxiety disorders and the onset of full AN is not supported [59]. The study of anxious temperament as a risk factor might reveal an interesting field of research beyond categorical diagnosis [60, 61]. The association between obsessive–compulsive disorder (OCD) and EDs, particularly AN, has been recognized for decades. The two disorders are risk factors for each other, with a lifetime prevalence of 13.9% in ED subjects [62]. Childhood OCD symptoms are more prevalent in individuals who develop EDs in adolescence, and some longitudinal data indicate childhood OCD symptoms as a risk factor, especially if the obsessions already concern food [63]. Neuroimaging studies reveal common, though non-specific, alterations in the anterior cingulate cortex in both disorders [64].

Limited evidence supports a bidirectional association between depressive symptoms and certain core ED symptoms (shape and weight dissatisfaction, overvaluation or preoccupation, dietary restraint, and binge eating) in adolescents [65]. One large sample study (*n* = 6361) found that psychotic experiences at the age of 13 years were associated with greater odds of reporting any disordered eating behaviors and more severe symptoms [66]. This is a relevant finding, given the known shared genetic vulnerability [54] and familial co-aggregation [67] of schizophrenia-spectrum psychosis and EDs, especially AN, as well as some phenomenological overlaps between eating symptoms and positive psychotic symptoms [68]. The link between attention–deficit hyperactivity disorder (ADHD) and autism spectrum disorders (ASD) with EDs remains, so far, suggestive but not established [69–71].

As for other more immediate precursors or correlates of ED symptoms, such as higher education attainment, use of appearance-focused social media, and body image-related factors, their role as predictors of a CHR–ED status is uncertain [53].

### Keys to early intervention in psychiatry

**Fig. 1:**
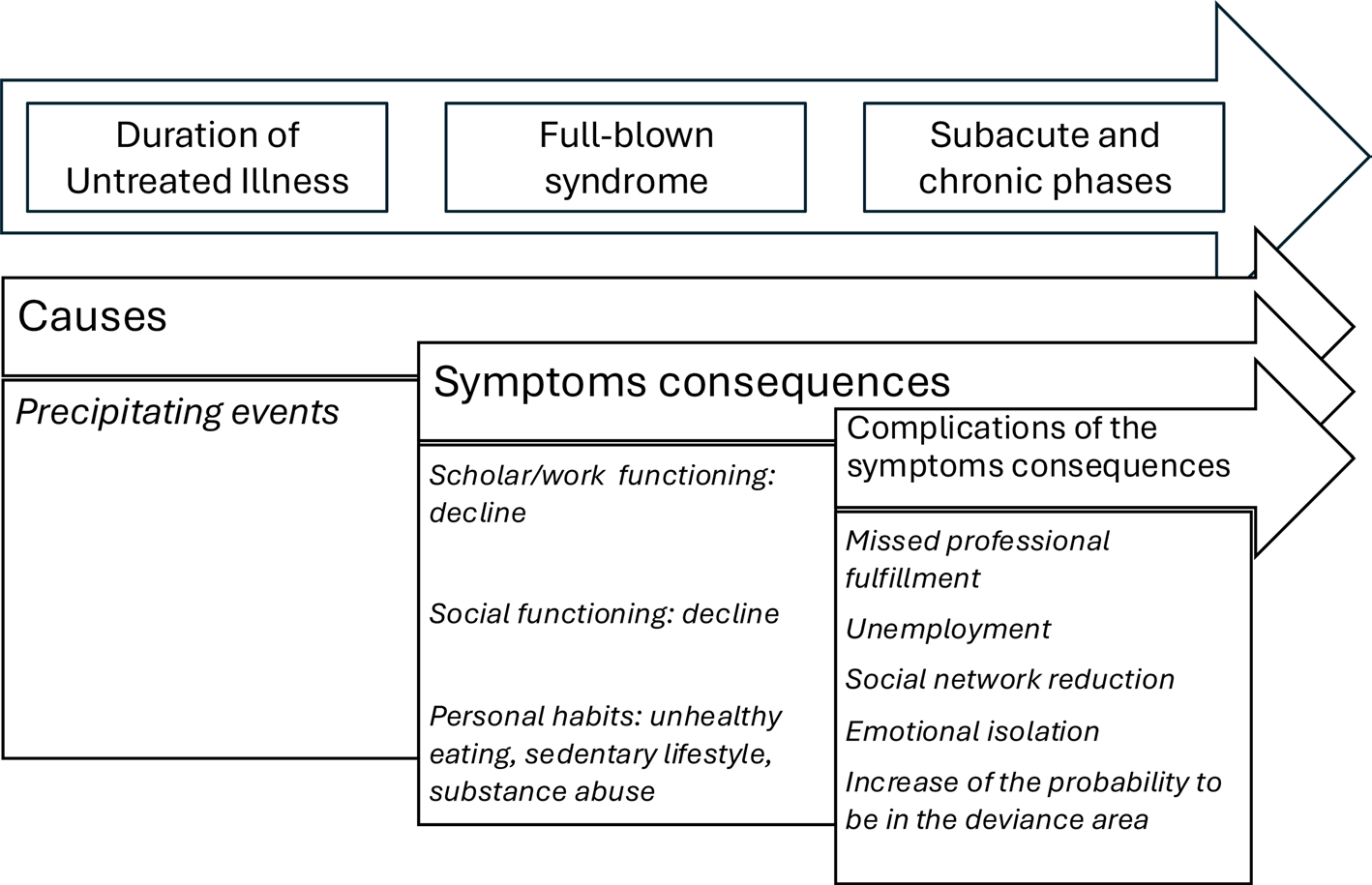
Medical model posits that symptoms of a mental disorder have a significant impact on psychosocial functioning and quality of life following its onset, further exacerbated by the negative consequences, sequelae, and complications of these symptoms. The graphic illustrates the main trajectories. The distal and proximate causes of eating disorders remain unknown; however, certain precipitating factors can often be identified and typically precede the onset of the full-blown syndrome. Early detection and treatment are critical. In the figure, the interval between the initial symptoms, linked to precipitating events, and the onset of the full-blown syndrome is depicted as a period of untreated illness. Once the syndrome develops, its symptoms lead to significant consequences affecting the individual's psychosocial adaptation and lifestyle. As the condition persists and progresses into subacute and chronic phases, complications from the symptoms and their sequelae compound the disorder's impact, potentially reducing the effectiveness of treatment. At this stage, addressing the core symptoms alone may no longer suffice to restore psychosocial and personal adaptation

Mental disorders may be conceived as the results of a long-term complex interaction of predisposing neurobiological vulnerabilities, including polygenic predisposition, and environmental risk factors. Of course, we can identify several antecedents and concomitants of the onset of a mental disorder, conceived as precipitating factors. After the onset of a mental disorder, several events contribute to its course and may influence its short- and long-term outcome (Fig. 1).

Time, indeed, is key in early intervention. Symptoms have direct consequences, since they impact social–occupational and relational functioning [72]. The impact of ongoing symptoms is as severe as long as they endure untreated. Moreover, many mental disorders begin in childhood and adolescence, and the disruption of primary social networks, such as schoolmates and sports friends, can hinder the social development of affected individuals. This disruption, coupled with the social cognition disabilities common in many mental disorders, can impair their capacity to navigate social rules [73–75]. Ongoing symptoms of a mental disorder may also influence lifestyle and impair health habits, increasing the chance of disordered eating, sedentary lifestyle, and alcohol and substance use [76].

When overlooked or unaddressed, early school leaving is an oft consequence of psychopathology, particularly when the disorder manifests early onset [77–80]. Academic underachievement frequently results in inadequate professional qualifications, thereby complicating job search endeavors and heightening the risk of underemployment and unemployment [81, 82]. Diminished socialization leads to smaller social networks, consequently reducing social support, elevating the risk of social isolation, and diminishing the likelihood of forming meaningful bonds [83]. When unhealthy lifestyle habits have been established, especially when a substance use disorder coexists, there is increased risk of somatic complications [84, 85], and secondary substance-related mental disorders may ensue. Avoiding the short- and long-term complications and sequelae of persisting, untreated psychological symptoms is the primary mission of early intervention in psychiatry.

### Prodromes: coordinates of a paradigm

Looking for the prodromes of an ED seems the best solution to avoid the worst consequences of untreated illness. After all, identifying a disorder at its very onset should allow early treatment, the main avenue to symptom remission and psychosocial recovery. Moreover, recognizing a disorder in its prodromal phase should facilitate the identification of etiologic factors and prevent its evolution toward chronicity and resistance to treatment. The definition of the prodromes of EDs is more easily attained within a staging approach. Indeed, the staging model enables clinicians to identify distinct disease phases and categorize patients based on established criteria. This approach can enhance treatment planning and the selection of targeted therapies, providing a framework for understanding longitudinal data on EDs in terms of relapse, recovery, and chronicity. So far, the main application of staging in EDs has been determining the longitudinal course of full syndromes, especially AN, focusing on criteria for remission and chronicity [86]. The creation of the harshly debated category of severe and enduring anorexia nervosa (SE–AN) is the main result of the staging approach in EDs [87, 88]. The definition of prodromes of EDs has remained theoretical or was based on the duration of the fully displayed syndrome [86, 89], and not on the symptoms profile in terms of severity, pervasiveness, and persistence, as in other fields of psychiatry [90]. Moreover, the identification of prodromes of EDs is challenging. The prodromal phase of an ED may be lengthy, and its detection is hampered by the non-specificity of most clinical manifestations that precede the onset of a fully displayed syndrome.

The act of dieting emerges as a prominent precursor to the development of an ED [91], particularly when coupled with extreme weight control strategies [92]. Dieting often precedes weight or shape concerns and may precipitate binge eating [92]. Additional known antecedents of an ED, such as anxiety, depression, social phobia and psychotic-like experiences might represent a risk factor, because they favor dieting or cause lack of appetence, which sometimes evolves into a rigid scheme of restrictive eating [59, 93, 94]. In this perspective, dietary restraint routines might exert anxiety reduction effects in individuals with preexisting high levels of anxiety [95, 96]. In some cases, however, anxiety follows the onset of an ED [59, 93]. Some personality traits, such as perfectionism for AN and impulsivity for BN and BED, may be involved in the onset and maintenance of the condition [97–102]. However, robust evidence is lacking [100, 103]. Accumulating evidence pinpoints early feeding and sleeping difficulties in childhood as the most remote antecedents and precursors of EDs in adolescence and adulthood [92].

Organizing such intricate and diverse arrays of factors into a cohesive framework poses challenges unless conceptualized within a neurodevelopmental model, where each element is integrated into a sequential phase.

### A neurodevelopmental perspective on mental disorders

In the offspring of parents with schizophrenia, longitudinal studies indicate that a subset of at-risk children exhibit early signs and markers of vulnerability, manifesting along deviant developmental trajectories well before the onset of psychosis since childhood premorbid stages [104]. Issues such as delayed motor development, language problems, deficits in general intelligence, sustained attention, executive functioning, and challenges in social cognition and adaptation are observed, with their occurrence appearing to align with the maturation sequence of activity patterns [105, 106]. This sequence initiates at primary sensory and motor functions in childhood and progresses to multisensory integration and higher cognitive functions during adolescence [107]. Both genetics [108] and epigenetics [109] factors are thought to influence the displaying of these abnormal patterns of maturation. A similar pattern, although less marked for severity and different for symptomatology has been robustly reported for offspring of parents with bipolar disorder [110, 111].

For EDs, there is no sequential maturation pattern as the one observed in schizophrenia spectrum disorders. This may depend on a lack of both retrospective and prospective longitudinal studies. Indeed, there is a dearth of longitudinal studies regarding EDs, and the available ones do not meet optimal quality standards [103]. Most robust findings derive from longitudinal studies of at-risk children, i.e., children of mothers with eating disorders, in particular AN [112]. Although disentangling genetic (e.g., polygenic vulnerability), organic (e.g., maternal eating behavior during pregnancy), and environmental factors (e.g., early obstetric complications and poor maternal feeding habits) in the transgenerational transmission of mental disorders, including EDs, is challenging [113], studies have identified certain patterns. At-risk children exhibit slightly different somatic growth in childhood [114, 115], early cognitive difficulties in infancy (e.g., social understanding, visual-motor function, planning, and abstract reasoning) that improve during schooling [116, 117], and broad psychopathological symptoms that vary by gender and are not strictly related to eating [118, 119].

Current literature does not permit delineate nor exclude that EDs and their prodromes related to eating are preceded by neurodevelopmental antecedents representing the putative premorbid vulnerability on which EDs emerge [120]. At the same time, some conditions (cognitive rigidity, perfectionism and childhood feeding disorders associated with both under or overweight) may be considered quite robust precursors, although their causal role in subsequent EDs is far from being fully understood. Other possible precursors, such as affective symptoms and psychotic-like experiences, are too broad to be considered as somehow specific premorbid manifestations.

### Chronopathogram: a tool to investigate the unfolding of symptoms across time in EDs

Expanding on previous research using validated medical history questionnaires on premorbid factors of EDs [121], we introduce a tool that may aid in precisely investigating the role of development in EDs. A chronopathogram is a graphical representation of pathological events as they unfold over time. It illustrates the temporal patterns of various aspects, such as symptoms or behaviors, related to a particular condition or disorder. The chronopathogram is used in neurology to represent the succession of crises in epilepsy. In psychiatry, it has been used to describe the succession over time of the episodes of depression and mania in patients with bipolar disorder. It is often used to depict fluctuations and rhythms over time. However, it can aid in defining the deployment of pathology across the different phases of life, especially in neurodevelopmental terms.

**Fig. 2:**
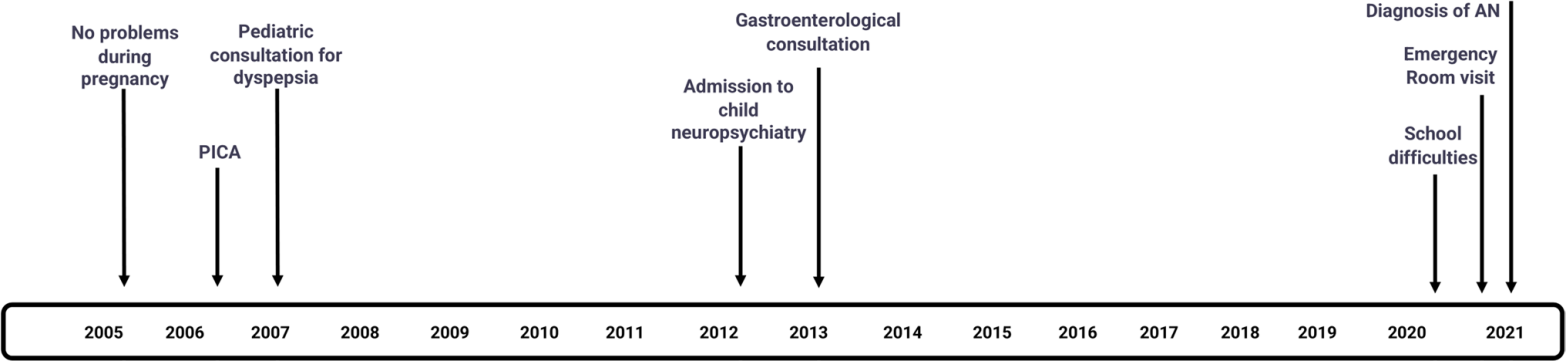
Chronopathogram of the emergence of symptoms before the onset of the full-blown condition in a case diagnosed with anorexia nervosa. The figure provides a retrospective synthesis of clinical events reconstructed from the anamnesis of a case of anorexia nervosa. The diagnosis was made in a 17-year-old girl during a psychiatric consultation requested by her parents following an emergency department visit due to concerns about significant weight loss amid prolonged school difficulties. In late childhood, she was admitted to a child and adolescent psychiatric service for unknown reasons and had a gastroenterological consultation for gastrointestinal issues. During infancy, she experienced a brief episode of pica and underwent a pediatric consultation for dyspepsia, though details are sparse. This information was chronologically organized into the chronopathogram, which synthesizes key events from ten cases to protect patient privacy by focusing only on the most significant details

We propose a version of the chronopathogram to be used to collect retrospectively and prospectively, depending on the design of the study, information about events and conditions that have preceded the diagnosis of an ED. In its simplest form, the tool annotates events of interest from pregnancy to diagnosis or a specific time (Fig. 2).

A more detailed acquisition of data can be achieved through a semi-structured interview. In the appendix, an interview is documented. The interview is designed to systematically collect data necessary for constructing a chronopathogram, a graphical representation of pathological events preceding the onset of EDs. It is structured to capture detailed information from caregivers regarding the subject’s developmental history, health events, and environmental factors. The rationale for the questions stems from the neurodevelopmental model proposed in the article, which emphasizes the importance of identifying antecedents, correlates, and triggers associated with ED onset. The interview is organized into thematic sections covering distinct life stages—pregnancy, infancy, childhood, and adolescence—allowing chronological mapping of key events. It begins with maternal factors during pregnancy and labor, including complications and exposures, as these may influence early vulnerability. Questions then progress to neonatal and early childhood health concerns, developmental milestones, and feeding behaviors, which align with known risk factors like picky eating or gastrointestinal problems. Finally, it explores psychosocial and behavioral issues during late childhood and adolescence, including anxiety, mood swings, and body image concerns, which are often precursors to EDs.

This structured approach ensures comprehensive data collection while respecting participant well-being, as caregivers can pause or seek clarification during the process. The chronopathogram synthesizes these findings, supporting retrospective and prospective analyses of ED trajectories.

## Conclusions

There is a significant need for new research and developments in ED treatment. Despite their greater prevalence and substantial burden and costs [4], EDs remain poorly studied compared to psychosis, as highlighted in a recent bibliometric study [122]. EDs could benefit greatly from early detection and intervention, but identifying prodromes is complex. Adopting a longitudinal approach appears particularly promising for early detection, as it allows for the observation of evolving patterns over time. Moreover, emphasizing neurodevelopmental perspectives proves valuable in discerning the antecedents, correlates, precipitants, and risk factors associated with EDs. Gathering data longitudinally enhances this endeavor, with tools like the chronopathogram proving instrumental in retrospectively capturing information from chart records and prospectively monitoring individuals at high risk for developing EDs.

### What is already known about this subject?

Timely identification and treatment of EDs can significantly improve prognosis. Although early intervention for EDs is still developing, it is clear that understanding risk factors is crucial due to challenges in identifying prodromal phases and measuring the duration of untreated illness.

### What does this study add?

This expert narrative review presents a neurodevelopmental model that integrates known risk factors for EDs. The model is designed to help identify the antecedents, concomitants, and precipitants of EDs. The article also presents a new tool: the chronopathogram, a graphical representation of pathological events, along with a semi-standardized interview designed to gather information about events that may have preceded, accompanied, or triggered an ED, which are then summarized in the chronopathogram.

## Supplementary Information

Below is the link to the electronic supplementary material.

Supplementary file 1.

## Data Availability

No datasets were generated or analysed during the current study.

## References

[1] Treasure J, Duarte TA, Schmidt U (2020) Eating disorders. The Lancet 395:899–911. 10.1016/S0140-6736(20)30059-3 10.1016/S0140-6736(20)30059-3 32171414

[2] American Psychiatric Association (2022) Diagnostic and statistical manual of mental disorders. American Psychiatric Association Publishing

[3] World Health Organization (2022) ICD-11: International classification of diseases (11th revision). In: https://icd.who.int/

[4] GBD 2019 Mental Disorders Collaborators (2022) Global, regional, and national burden of 12 mental disorders in 204 countries and territories, 1990–2019: a systematic analysis for the Global Burden of Disease Study 2019. Lancet Psychiatry 9:137–150. 10.1016/S2215-0366(21)00395-3 10.1016/S2215-0366(21)00395-3 PMC8776563 35026139

[5] van Hoeken D, Hoek HW (2020) Review of the burden of eating disorders: mortality, disability, costs, quality of life, and family burden. Curr Opin Psychiatry 33:521–527. 10.1097/YCO.0000000000000641 32796186 10.1097/YCO.0000000000000641 PMC7575017

[6] Galmiche M, Déchelotte P, Lambert G, Tavolacci MP (2019) Prevalence of eating disorders over the 2000–2018 period: a systematic literature review. Am J Clin Nutr 109:1402–1413. 10.1093/ajcn/nqy342 31051507 10.1093/ajcn/nqy342

[7] Silén Y, Keski-Rahkonen A (2022) Worldwide prevalence of DSM-5 eating disorders among young people. Curr Opin Psychiatry 35:362–371. 10.1097/YCO.0000000000000818 36125216 10.1097/YCO.0000000000000818

[8] Panero M, Abbate-Daga G (2023) What’s new in research during the current epidemic wave of eating disorders? J Clin Med 12:3994. 10.3390/jcm12123994 37373686 10.3390/jcm12123994 PMC10299221

[9] Güzel Â, Mutlu NL, Molendijk M (2023) COVID-19-related changes in eating disorder pathology, emotional and binge eating and need for care: a systematic review with frequentist and Bayesian meta-analyses. Eat Weight Disord Stud Anorex Bulim Obes 28:19. 10.1007/s40519-023-01547-2 10.1007/s40519-023-01547-2 PMC9941242 36805344

[10] Cassioli E, Sensi C, Mannucci E et al (2020) Pharmacological treatment of acute-phase anorexia nervosa: evidence from randomized controlled trials. J Psychopharmacol 34:864–873. 10.1177/0269881120920453 32448045 10.1177/0269881120920453

[11] Monteleone AM, Pellegrino F, Croatto G et al (2022) Treatment of eating disorders: a systematic meta-review of meta-analyses and network meta-analyses. Neurosci Biobehav Rev 142:104857. 10.1016/j.neubiorev.2022.104857 36084848 10.1016/j.neubiorev.2022.104857 PMC9813802

[12] Argyrou A, Lappas AS, Bakaloudi DR et al (2023) Pharmacotherapy compared to placebo for people with Bulimia Nervosa: a systematic review and meta-analysis. Psychiatry Res 327:115357. 10.1016/j.psychres.2023.115357 37562154 10.1016/j.psychres.2023.115357

[13] Milano W, Capasso A (2019) Psychopharmacological options in the multidisciplinary and multidimensional treatment of eating disorders. Open Neurol J 13:22–31. 10.2174/1874205X01913010022

[14] Solmi M, Radua J, Stubbs B et al (2021) Risk factors for eating disorders: an umbrella review of published meta-analyses. Braz J Psychiatry 43:314–323. 10.1590/1516-4446-2020-1099 32997075 10.1590/1516-4446-2020-1099 PMC8136381

[15] Grilo CM, Juarascio A (2023) Binge-eating disorder interventions: review, current status, and implications. Curr Obes Rep 12:406–416. 10.1007/s13679-023-00517-0 37439970 10.1007/s13679-023-00517-0 PMC10528223

[16] Murray SB, Strober M, Tadayonnejad R et al (2022) Neurosurgery and neuromodulation for anorexia nervosa in the 21st century: a systematic review of treatment outcomes. Eat Disord 30:26–53. 10.1080/10640266.2020.1790270 32991247 10.1080/10640266.2020.1790270 PMC8386186

[17] McClelland J, Bozhilova N, Campbell I, Schmidt U (2013) A systematic review of the effects of neuromodulation on eating and body weight: evidence from human and animal studies. Eur Eat Disord Rev 21:436–455. 10.1002/erv.2256 24155246 10.1002/erv.2256

[18] Phillipou A, McGorry P, Killackey E, Maguire S (2023) Eating disorders in young people. Australas Psychiatry 31:346–348. 10.1177/10398562231159514 36853994 10.1177/10398562231159514

[19] Tiller J, Maguire T, Newman-Taylor K (2023) Early intervention in psychosis services: a systematic review and narrative synthesis of barriers and facilitators to seeking access. Eur Psychiatry 66:e92. 10.1192/j.eurpsy.2023.2465 37929296 10.1192/j.eurpsy.2023.2465 PMC10755576

[20] Crisp AH (1979) Early recognition and prevention of Anorexia Nervosa. Dev Med Child Neurol 21:393–395. 10.1111/j.1469-8749.1979.tb01634.x 467823 10.1111/j.1469-8749.1979.tb01634.x

[21] Herzog DB (1982) Bulimia in the adolescent. Arch Pediatr Adolesc Med 136:985. 10.1001/archpedi.1982.03970470029007 10.1001/archpedi.1982.03970470029007 6957146

[22] Johnson M (1982) Anorexia nervosa: framework for early identification and intervention. Issues Ment Health Nurs 4:87–99. 10.3109/01612848209141046 6924653 10.3109/01612848209141046

[23] Schoemaker C (1997) Does early intervention improve the prognosis in anorexia nervosa? A systematic review of the treatment-outcome literature. Int J Eat Disord 21:1–15. 10.1002/(SICI)1098-108X(199701)21:1%3c1::AID-EAT1%3e3.0.CO;2-R 8986512 10.1002/(sici)1098-108x(199701)21:1<1::aid-eat1>3.0.co;2-r

[24] Currin L, Schmidt U (2005) A critical analysis of the utility of an early intervention approach in the eating disorders. J Ment Health 14:611–624. 10.1080/09638230500347939

[25] Treasure J, Stein D, Maguire S (2015) Has the time come for a staging model to map the course of eating disorders from high risk to severe enduring illness? An examination of the evidence. Early Interv Psychiatry 9:173–184. 10.1111/eip.12170 25263388 10.1111/eip.12170

[26] Radunz M, Keegan E, Osenk I, Wade TD (2020) Relationship between eating disorder duration and treatment outcome: systematic review and meta-analysis. Int J Eat Disord 53:1761–1773. 10.1002/eat.23373 32856329 10.1002/eat.23373

[27] Gumz A, Reuter L, Löwe B et al (2023) Factors influencing the duration of untreated illness among patients with anorexia nervosa <scp>: a</scp> multicenter and multi-informant study. Int J Eat Disord 56:2315–2327. 10.1002/eat.24069 37814447 10.1002/eat.24069

[28] Vandereycken W (2006) Denial of illness in anorexia nervosa—a conceptual review: part 2 different forms and meanings. Eur Eat Disord Rev 14:352–368. 10.1002/erv.722

[29] Radunz M, Ali K, Wade TD (2023) Pathways to improve early intervention for eating disorders: Findings from a systematic review and meta-analysis. Int J Eat Disord 56:314–330. 10.1002/eat.23845 36346008 10.1002/eat.23845

[30] Ali K, Farrer L, Fassnacht DB et al (2017) Perceived barriers and facilitators towards help-seeking for eating disorders: a systematic review. Int J Eat Disord 50:9–21. 10.1002/eat.22598 27526643 10.1002/eat.22598

[31] Abbate-Daga G, Amianto F, Delsedime N et al (2013) Resistance to treatment and change in anorexia nervosa: a clinical overview. BMC Psychiatry 13:294. 10.1186/1471-244X-13-294 24199620 10.1186/1471-244X-13-294 PMC3879222

[32] Gregertsen EC, Mandy W, Serpell L (2017) The egosyntonic nature of anorexia: an impediment to recovery in anorexia nervosa treatment. Front Psychol. 10.3389/fpsyg.2017.02273 29312100 10.3389/fpsyg.2017.02273 PMC5743910

[33] Nagy H, Paul T, Jain E et al (2023) A clinical overview of anorexia nervosa and overcoming treatment resistance. Avicenna J Med 13:003–014. 10.1055/s-0042-1758859 10.1055/s-0042-1758859 PMC10038755 36969350

[34] Monteleone AM, Barone E, Cascino G et al (2023) Pathways to eating disorder care: a European multicenter study. Eur Psychiatry 66:e36. 10.1192/j.eurpsy.2023.23 37092677 10.1192/j.eurpsy.2023.23 PMC10228357

[35] Hamilton A, Mitchison D, Basten C et al (2022) Understanding treatment delay: perceived barriers preventing treatment-seeking for eating disorders. Aust N Z J Psychiatry 56:248–259. 10.1177/00048674211020102 34250844 10.1177/00048674211020102

[36] Yeo M, Hughes E (2011) Eating disorders-early identification in general practice. Aust Fam Physician 40:108–111 21597510

[37] Wells KR, Jeacocke NA, Appaneal R et al (2020) The Australian Institute of Sport (AIS) and National Eating Disorders Collaboration (NEDC) position statement on disordered eating in high performance sport. Br J Sports Med 54:1247–1258. 10.1136/bjsports-2019-101813 32661127 10.1136/bjsports-2019-101813 PMC7588409

[38] Anderson KK, Norman R, MacDougall A et al (2018) Effectiveness of early psychosis intervention: comparison of service users and nonusers in population-based health administrative data. Am J Psychiatry 175:443–452. 10.1176/appi.ajp.2017.17050480 29495897 10.1176/appi.ajp.2017.17050480

[39] Malla A, McGorry P (2019) Early intervention in psychosis in young people: a population and public health perspective. Am J Public Health 109:S181–S184. 10.2105/AJPH.2019.305018 31242015 10.2105/AJPH.2019.305018 PMC6595512

[40] Austin A, Flynn M, Richards K et al (2021) Duration of untreated eating disorder and relationship to outcomes: a systematic review of the literature. Eur Eat Disord Rev 29:329–345. 10.1002/erv.2745 32578311 10.1002/erv.2745

[41] Ranta K, Väänänen J, Fröjd S et al (2017) Social phobia, depression and eating disorders during middle adolescence: longitudinal associations and treatment seeking. Nord J Psychiatry 71:605–613. 10.1080/08039488.2017.1366548 28868945 10.1080/08039488.2017.1366548

[42] Schaumberg K, Zerwas S, Goodman E et al (2019) Anxiety disorder symptoms at age 10 predict eating disorder symptoms and diagnoses in adolescence. J Child Psychol Psychiatry 60:686–696. 10.1111/jcpp.12984 30353925 10.1111/jcpp.12984 PMC6482103

[43] Stice E, Desjardins CD, Rohde P, Shaw H (2021) Sequencing of symptom emergence in anorexia nervosa, bulimia nervosa, binge eating disorder, and purging disorder and relations of prodromal symptoms to future onset of these disorders. J Abnorm Psychol 130:377–387. 10.1037/abn0000666 34180702 10.1037/abn0000666 PMC8244173

[44] Yamamiya Y, Desjardins CD, Stice E (2023) Sequencing of symptom emergence in anorexia nervosa, bulimia nervosa, binge eating disorder, and purging disorder in adolescent girls and relations of prodromal symptoms to future onset of these eating disorders. Psychol Med 53:4657–4665. 10.1017/S0033291722001568 37698515 10.1017/S0033291722001568

[45] Christian C, Perko VL, Vanzhula IA et al (2020) Eating disorder core symptoms and symptom pathways across developmental stages: a network analysis. J Abnorm Psychol 129:177–190. 10.1037/abn0000477 31714097 10.1037/abn0000477

[46] Allen KL, Mountford VA, Elwyn R et al (2023) A framework for conceptualising early intervention for eating disorders. Eur Eat Disord Rev 31:320–334. 10.1002/erv.2959 36426567 10.1002/erv.2959 PMC10100476

[47] Rienecke R (2017) Family-based treatment of eating disorders in adolescents: current insights. Adolesc Health Med Ther 8:69–79. 10.2147/AHMT.S115775 28615982 10.2147/AHMT.S115775 PMC5459462

[48] Loeb KL, Weissman RS, Marcus S et al (2020) Family-based treatment for anorexia nervosa symptoms in high-risk youth: a partially-randomized preference-design study. Front Psychiatry. 10.3389/fpsyt.2019.00985 32038326 10.3389/fpsyt.2019.00985 PMC6987468

[49] Brown A, McClelland J, Boysen E et al (2018) The FREED Project (first episode and rapid early intervention in eating disorders): service model, feasibility and acceptability. Early Interv Psychiatry 12:250–257. 10.1111/eip.12382 27619198 10.1111/eip.12382

[50] Austin A, Flynn M, Shearer J et al (2022) The first episode rapid early intervention for eating disorders-upscaled study: clinical outcomes. Early Interv Psychiatry 16:97–105. 10.1111/eip.13139 33781000 10.1111/eip.13139 PMC9291113

[51] Murray SB, Quintana DS, Loeb KL et al (2019) Treatment outcomes for anorexia nervosa: a systematic review and meta-analysis of randomized controlled trials. Psychol Med 49:535–544. 10.1017/S0033291718002088 30101734 10.1017/S0033291718002088

[52] Jacobi C, Hayward C, de Zwaan M et al (2004) Coming to terms with risk factors for eating disorders: application of risk terminology and suggestions for a general taxonomy. Psychol Bull 130:19–65. 10.1037/0033-2909.130.1.19 14717649 10.1037/0033-2909.130.1.19

[53] Barakat S, McLean SA, Bryant E et al (2023) Risk factors for eating disorders: findings from a rapid review. J Eat Disord 11:8 36650572 10.1186/s40337-022-00717-4 PMC9847054

[54] Watson HJ, Palmos AB, Hunjan A et al (2021) Genetics of eating disorders in the genome-wide era. Psychol Med 51:2287–2297. 10.1017/S0033291720005474 33583449 10.1017/S0033291720005474 PMC8790815

[55] Bulik CM, Coleman JRI, Hardaway JA et al (2022) Genetics and neurobiology of eating disorders. Nat Neurosci 25:543–554. 10.1038/s41593-022-01071-z 35524137 10.1038/s41593-022-01071-z PMC9744360

[56] de Jorge MC, Rukh G, Williams MJ et al (2022) Genetics of anorexia nervosa: an overview of genome-wide association studies and emerging biological links. J Genet Genomics 49:1–12. 10.1016/j.jgg.2021.09.005 34634498 10.1016/j.jgg.2021.09.005

[57] Longo P, Bertorello A, Panero M et al (2019) (2019) Traumatic events and post-traumatic symptoms in anorexia nervosa. Eur J Psychotraumatol. 10.1080/20008198.2019.1682930 31723378 10.1080/20008198.2019.1682930 PMC6830292

[58] Hayes S, Linardon J, Kim C, Mitchison D (2021) Understanding the relationship between sexual harassment and eating disorder psychopathology: a systematic review and meta-analysis. Int J Eat Disord 54:673–689. 10.1002/eat.23499 33751633 10.1002/eat.23499

[59] Lloyd EC, Haase AM, Foster CE, Verplanken B (2019) A systematic review of studies probing longitudinal associations between anxiety and anorexia nervosa. Psychiatry Res 276:175–185. 10.1016/j.psychres.2019.05.010 31096148 10.1016/j.psychres.2019.05.010

[60] Marzola E, Porliod A, Panero M et al (2020) Affective temperaments and eating psychopathology in anorexia nervosa: Which role for anxious and depressive traits? J Affect Disord 266:374–380. 10.1016/j.jad.2020.01.142 32056902 10.1016/j.jad.2020.01.142

[61] Favaretto E, Bedani F, Brancati GE et al (2024) Synthesising 30 years of clinical experience and scientific insight on affective temperaments in psychiatric disorders: state of the art. J Affect Disord 362:406–415. 10.1016/j.jad.2024.07.011 38972642 10.1016/j.jad.2024.07.011

[62] Drakes DH, Fawcett EJ, Rose JP et al (2021) Comorbid obsessive-compulsive disorder in individuals with eating disorders: an epidemiological meta-analysis. J Psychiatr Res 141:176–191. 10.1016/j.jpsychires.2021.06.035 34216946 10.1016/j.jpsychires.2021.06.035

[63] Micali N, Hilton K, Natatani E et al (2011) Is childhood OCD a risk factor for eating disorders later in life? A longitudinal study. Psychol Med 41:2507–2513. 10.1017/S003329171100078X 21733209 10.1017/S003329171100078X

[64] Di Luzio M, Bellantoni D, Bellantoni AL et al (2024) Similarities and differences between eating disorders and obsessive-compulsive disorder in childhood and adolescence: a systematic review. Front Psychiatry. 10.3389/fpsyt.2024.1407872 38895032 10.3389/fpsyt.2024.1407872 PMC11183500

[65] Kenny B, Fuller-Tyszkiewicz M, Moodie M et al (2022) Bi-directional associations between depressive symptoms and eating disorder symptoms in early adolescence. Body Image 42:246–256. 10.1016/j.bodyim.2022.06.012 35841698 10.1016/j.bodyim.2022.06.012

[66] Solmi F, Melamed D, Lewis G, Kirkbride JB (2018) Longitudinal associations between psychotic experiences and disordered eating behaviours in adolescence: a UK population-based study. Lancet Child Adolesc Health 2:591–599. 10.1016/S2352-4642(18)30180-9 30119718 10.1016/S2352-4642(18)30180-9 PMC6054050

[67] Zhang R, Larsen JT, Kuja-Halkola R et al (2021) Familial co-aggregation of schizophrenia and eating disorders in Sweden and Denmark. Mol Psychiatry 26:5389–5397. 10.1038/s41380-020-0749-x 32382133 10.1038/s41380-020-0749-x

[68] Poletti M, Preti A, Raballo A (2022) Eating disorders and psychosis as intertwined dimensions of disembodiment: a narrative review. Clin Neuropsychiatry 19:187–192. 10.36131/cnfioritieditore20220307 35821871 10.36131/cnfioritieditore20220307 PMC9263683

[69] Huke V, Turk J, Saeidi S et al (2013) Autism spectrum disorders in eating disorder populations: a systematic review. Eur Eat Disord Rev 21:345–351. 10.1002/erv.2244 23900859 10.1002/erv.2244

[70] Nazar BP, Bernardes C, Peachey G et al (2016) The risk of eating disorders comorbid with attention-deficit/hyperactivity disorder: a systematic review and meta-analysis. Int J Eat Disord 49:1045–1057. 10.1002/eat.22643 27859581 10.1002/eat.22643

[71] Saure E, Laasonen M, Lepistö-Paisley T et al (2020) Characteristics of autism spectrum disorders are associated with longer duration of anorexia nervosa: a systematic review and meta-analysis. Int J Eat Disord 53:1056–1079. 10.1002/eat.23259 32181530 10.1002/eat.23259

[72] Ro E, Clark LA (2009) Psychosocial functioning in the context of diagnosis: assessment and theoretical issues. Psychol Assess 21:313–324. 10.1037/a0016611 19719344 10.1037/a0016611

[73] Mier D, Kirsch P (2015) Social-Cognitive Deficits in Schizophrenia. pp 397–409 10.1007/7854_2015_427 26728167

[74] Gillissie ES, Lui LMW, Ceban F et al (2022) Deficits of social cognition in bipolar disorder: systematic review and meta-analysis. Bipolar Disord 24:137–148. 10.1111/bdi.13163 34825440 10.1111/bdi.13163

[75] Tauro JL, Wearne TA, Belevski B et al (2022) Social cognition in female adults with Anorexia Nervosa: a systematic review. Neurosci Biobehav Rev 132:197–210. 10.1016/j.neubiorev.2021.11.035 34822877 10.1016/j.neubiorev.2021.11.035

[76] Cabassa LJ, Ezell JM, Lewis-Fernández R (2010) Lifestyle interventions for adults with serious mental illness: a systematic literature review. Psychiatr Serv 61:774–782. 10.1176/ps.2010.61.8.774 20675835 10.1176/appi.ps.61.8.774 PMC3632414

[77] Esch P, Bocquet V, Pull C et al (2014) The downward spiral of mental disorders and educational attainment: a systematic review on early school leaving. BMC Psychiatry 14:237. 10.1186/s12888-014-0237-4 25159271 10.1186/s12888-014-0237-4 PMC4244046

[78] Dickson H, Hedges EP, Ma SY et al (2020) Academic achievement and schizophrenia: a systematic meta-analysis. Psychol Med 50:1949–1965. 10.1017/S0033291720002354 32684198 10.1017/S0033291720002354

[79] Agnafors S, Barmark M, Sydsjö G (2024) Correction: mental health and academic performance: a study on selection and causation effects from childhood to early adulthood. Soc Psychiatry Psychiatr Epidemiol 59:199–199. 10.1007/s00127-023-02560-7 37715813 10.1007/s00127-023-02560-7 PMC10799831

[80] Crossley NA, Alliende LM, Czepielewski LS et al (2022) The enduring gap in educational attainment in schizophrenia according to the past 50 years of published research: a systematic review and meta-analysis. Lancet Psychiatry 9:565–573. 10.1016/S2215-0366(22)00121-3 35717966 10.1016/S2215-0366(22)00121-3

[81] Helgesson M, Tinghög P, Wang M et al (2018) Trajectories of work disability and unemployment among young adults with common mental disorders. BMC Public Health 18:1228. 10.1186/s12889-018-6141-y 30400785 10.1186/s12889-018-6141-y PMC6219052

[82] Mousteri V, Daly M, Delaney L et al (2019) Adolescent mental health and unemployment over the lifespan: population evidence from Sweden. Soc Sci Med 222:305–314. 10.1016/j.socscimed.2018.12.030 30677644 10.1016/j.socscimed.2018.12.030

[83] Cloutier B, Francoeur A, Samson C et al (2021) Romantic relationships, sexuality, and psychotic disorders: a systematic review of recent findings. Psychiatr Rehabil J 44:22–42. 10.1037/prj0000409 32191102 10.1037/prj0000409

[84] Filipčić I, Šimunović Filipčić I, Grošić V et al (2018) Patterns of chronic physical multimorbidity in psychiatric and general population. J Psychosom Res 114:72–80. 10.1016/j.jpsychores.2018.09.011 30314582 10.1016/j.jpsychores.2018.09.011

[85] Firth J, Siddiqi N, Koyanagi A et al (2019) The lancet psychiatry commission: a blueprint for protecting physical health in people with mental illness. Lancet Psychiatry 6:675–712. 10.1016/S2215-0366(19)30132-4 31324560 10.1016/S2215-0366(19)30132-4

[86] Tomba E, Tecuta L, Gardini V et al (2024) Staging models in eating disorders: a systematic scoping review of the literature. Compr Psychiatry 131:152468. 10.1016/j.comppsych.2024.152468 38460478 10.1016/j.comppsych.2024.152468

[87] Broomfield C, Stedal K, Touyz S, Rhodes P (2017) Labeling and defining severe and enduring anorexia nervosa: a systematic review and critical analysis. Int J Eat Disord 50:611–623. 10.1002/eat.22715 28444828 10.1002/eat.22715

[88] Voswinkel MM, Hanegraaff SM, Mares SHW et al (2024) Ethical implications of defining longstanding anorexia nervosa. J Eat Disord 12:77. 10.1186/s40337-024-01040-w 38863013 10.1186/s40337-024-01040-w PMC11165790

[89] Ambwani S, Cardi V, Albano G et al (2020) A multicenter audit of outpatient care for adult anorexia nervosa: symptom trajectory, service use, and evidence in support of “early stage” versus “severe and enduring” classification. Int J Eat Disord 53:1337–1348. 10.1002/eat.23246 32064663 10.1002/eat.23246

[90] Scott J, Iorfino F, Capon W et al (2024) Staging 2.0: refining transdiagnostic clinical staging frameworks to enhance reliability and utility for youth mental health. Lancet Psychiatry 11:461–471. 10.1016/S2215-0366(24)00060-9 38643773 10.1016/S2215-0366(24)00060-9

[91] Patton GC (1992) Eating disorders: antecedents, evolution and course. Ann Med 24:281–285. 10.3109/07853899209149955 1389090 10.3109/07853899209149955

[92] McClelland J, Robinson L, Potterton R et al (2020) Symptom trajectories into eating disorders: a systematic review of longitudinal, nonclinical studies in children/adolescents. Eur Psychiatry 63:e60. 10.1192/j.eurpsy.2020.55 32450945 10.1192/j.eurpsy.2020.55 PMC7355161

[93] Kerr-Gaffney J, Harrison A, Tchanturia K (2018) Social anxiety in the eating disorders: a systematic review and meta-analysis. Psychol Med 48:2477–2491. 10.1017/S0033291718000752 29631640 10.1017/S0033291718000752

[94] Garcia SC, Mikhail ME, Keel PK et al (2020) Increased rates of eating disorders and their symptoms in women with major depressive disorder and anxiety disorders. Int J Eat Disord 53:1844–1854. 10.1002/eat.23366 32844425 10.1002/eat.23366 PMC7669595

[95] Pallister E, Waller G (2008) Anxiety in the eating disorders: understanding the overlap. Clin Psychol Rev 28:366–386. 10.1016/j.cpr.2007.07.001 17707562 10.1016/j.cpr.2007.07.001

[96] Schaumberg K, Reilly EE, Gorrell S et al (2021) Conceptualizing eating disorder psychopathology using an anxiety disorders framework: evidence and implications for exposure-based clinical research. Clin Psychol Rev 83:101952. 10.1016/j.cpr.2020.101952 33221621 10.1016/j.cpr.2020.101952 PMC7868093

[97] Carr MM, Wiedemann AA, Macdonald-Gagnon G, Potenza MN (2021) Impulsivity and compulsivity in binge eating disorder: a systematic review of behavioral studies. Prog Neuropsychopharmacol Biol Psychiatry 110:110318. 10.1016/j.pnpbp.2021.110318 33794320 10.1016/j.pnpbp.2021.110318 PMC8222068

[98] Dahlenburg SC, Gleaves DH, Hutchinson AD (2019) Anorexia nervosa and perfectionism: a meta-analysis. Int J Eat Disord 52:219–229 30632629 10.1002/eat.23009

[99] Halmi KA, Bellace D, Berthod S et al (2012) An examination of early childhood perfectionism across anorexia nervosa subtypes. Int J Eat Disord 45:800–807. 10.1002/eat.22019 22488115 10.1002/eat.22019 PMC3418385

[100] Howard M, Gregertsen EC, Hindocha C, Serpell L (2020) Impulsivity and compulsivity in anorexia and bulimia nervosa: a systematic review. Psychiatry Res 293:113354. 10.1016/j.psychres.2020.113354 32781364 10.1016/j.psychres.2020.113354

[101] Longo P, Bevione F, Amodeo L et al (2024) Perfectionism in anorexia nervosa: associations with clinical picture and personality traits. Clin Psychol Psychother. 10.1002/cpp.2931 10.1002/cpp.2931 37970961

[102] Machado BC, Gonçalves SF, Martins C et al (2014) Risk factors and antecedent life events in the development of anorexia nervosa: a portuguese case-control study. Eur Eat Disord Rev 22:243–251. 10.1002/erv.2286 24577737 10.1002/erv.2286

[103] Charrat J-P, Massoubre C, Germain N et al (2023) Systematic review of prospective studies assessing risk factors to predict anorexia nervosa onset. J Eat Disord 11:163. 10.1186/s40337-023-00882-0 37730675 10.1186/s40337-023-00882-0 PMC10510169

[104] Duffy A, Goodday SM, Christiansen H et al (2023) The well-being of children at familial risk of severe mental illness: an overlooked yet crucial prevention and early intervention opportunity. Nature Mental Health 1:534–541. 10.1038/s44220-023-00090-4

[105] Hameed MA, Lewis AJ (2016) Offspring of parents with schizophrenia. Harv Rev Psychiatry 24:104–117. 10.1097/HRP.0000000000000076 26954595 10.1097/HRP.0000000000000076

[106] Poletti M, Raballo A (2020) Developmental psychotic risk: toward a neurodevelopmentally informed staging of vulnerability to psychosis. Harv Rev Psychiatry 28:271–278. 10.1097/HRP.0000000000000266 32692090 10.1097/HRP.0000000000000266

[107] Forsyth JK, Bearden CE (2023) Rethinking the first episode of schizophrenia: identifying convergent mechanisms during development and moving toward prediction. Am J Psychiatry 180:792–804. 10.1176/appi.ajp.20230736 37908094 10.1176/appi.ajp.20230736

[108] Legge SE, Santoro ML, Periyasamy S et al (2021) Genetic architecture of schizophrenia: a review of major advancements. Psychol Med 51:2168–2177. 10.1017/S0033291720005334 33550997 10.1017/S0033291720005334

[109] Davies C, Segre G, Estradé A et al (2020) Prenatal and perinatal risk and protective factors for psychosis: a systematic review and meta-analysis. Lancet Psychiatry 7:399–410. 10.1016/S2215-0366(20)30057-2 32220288 10.1016/S2215-0366(20)30057-2

[110] Duffy A, Goodday S, Keown-Stoneman C, Grof P (2019) The emergent course of bipolar disorder: observations over two decades from the canadian high-risk offspring cohort. Am J Psychiatry 176:720–729. 10.1176/appi.ajp.2018.18040461 30525908 10.1176/appi.ajp.2018.18040461

[111] Parellada M, Gomez-Vallejo S, Burdeus M, Arango C (2017) Developmental differences between schizophrenia and bipolar disorder. Schizophr Bull 43:1176–1189. 10.1093/schbul/sbx126 29045744 10.1093/schbul/sbx126 PMC5737496

[112] Mah B, Cibralic S, Hanna J et al (2021) Outcomes for infants whose mothers had an eating disorder in the perinatal period: a systematic review of the evidence. Int J Eat Disord 54:2077–2094. 10.1002/eat.23612 34608655 10.1002/eat.23612

[113] Raballo A, Poletti M, Preti A (2021) Applying transgenerational scientific evidence to the next wave of early identification strategies for psychopathological risk—transdiagnostic, developmental, and personalized. JAMA Psychiat 78:1067. 10.1001/jamapsychiatry.2021.1901 10.1001/jamapsychiatry.2021.1901 34347022

[114] Easter A, Howe LD, Tilling K et al (2014) Growth trajectories in the children of mothers with eating disorders: a longitudinal study. BMJ Open 4:e004453. 10.1136/bmjopen-2013-004453 24674996 10.1136/bmjopen-2013-004453 PMC3975767

[115] Solmi F, Sallis H, Stahl D et al (2014) Low birth weight in the offspring of women with anorexia nervosa. Epidemiol Rev 36:49–56. 10.1093/epirev/mxt004 24025351 10.1093/epirev/mxt004 PMC3873840

[116] Kothari R, Solmi F, Treasure J, Micali N (2013) The neuropsychological profile of children at high risk of developing an eating disorder. Psychol Med 43:1543–1554. 10.1017/S0033291712002188 23021014 10.1017/S0033291712002188

[117] Kothari R, Rosinska M, Treasure J, Micali N (2014) The early cognitive development of children at high risk of developing an eating disorder. Eur Eat Disord Rev 22:152–156. 10.1002/erv.2274 24375832 10.1002/erv.2274 PMC4208682

[118] Mantel Ä, Örtqvist AK, Hirschberg AL, Stephansson O (2022) Analysis of neurodevelopmental disorders in offspring of mothers with eating disorders in Sweden. JAMA Netw Open 5:e2143947. 10.1001/jamanetworkopen.2021.43947 35040968 10.1001/jamanetworkopen.2021.43947 PMC8767445

[119] Micali N, Stahl D, Treasure J, Simonoff E (2014) Childhood psychopathology in children of women with eating disorders: understanding risk mechanisms. J Child Psychol Psychiatry 55:124–134. 10.1111/jcpp.12112 23808622 10.1111/jcpp.12112 PMC4217387

[120] Marzola E, Cavallo F, Panero M et al (2021) The role of prenatal and perinatal factors in eating disorders: a systematic review. Arch Womens Ment Health 24:185–204. 10.1007/s00737-020-01057-5 32767123 10.1007/s00737-020-01057-5 PMC7979621

[121] Marzola E, Fassino S, Migliaretti G et al (2019) Development and validation of the premorbid childhood traits questionnaire (PCT-Q) in eating disorders. Eat Weight Disord Stud Anorexia Bulim Obes 24:815–823. 10.1007/s40519-019-00748-y 10.1007/s40519-019-00748-y 31313252

[122] Marzola E, Panero M, Longo P et al (2022) Research in eating disorders: the misunderstanding of supposing serious mental illnesses as a niche specialty. Eat Weight Disord Stud Anorexia, Bulim Obes 27:3005–3016. 10.1007/s40519-022-01473-9 10.1007/s40519-022-01473-9 PMC9462607 36085407

