# Supplementary material for: Early intervention in eating disorders: introducing the chronopathogram
Source: Eat Weight Disord. 2025 Jan 23;30(1):9. doi: 10.1007/s40519-025-01715-6 (PMC11759457; doi:10.1007/s40519-025-01715-6)
Supplement: Supplementary file 1 [file 40519_2025_1715_MOESM1_ESM.pdf]

Appendix

I would like to ask you some questions about the health of your daughter/son. These questions concern the pregnancy, early infancy, childhood, and adolescence until your daughter/son has received the diagnosis of an eating disorder. Feel free to ask me anything about these questions. You can interrupt this interview whenever you feel distressed.

How old were you when you became pregnant with [name of the patient with the eating disorder]?	____________________
Was this your first pregnancy?	☐ Yes ☐ No
How many before?	____________________
Did you have any problems during the pregnancy?	☐ Yes ☐ No
[If yes], When the problem did occur, which trimester?	☐ First ☐ Second ☐ Third
Did you take any drugs during the pregnancy?	☐ Yes ☐ No
Which drug did you take and in which trimester?	____________________ ☐ First ☐ Second ☐ Third<br>____________________ ☐ First ☐ Second ☐ Third<br>____________________ ☐ First ☐ Second ☐ Third
Did you have any troubles during the labor?	☐ Yes ☐ No
[If yes], Which kind of trouble?	____________________
Did your daughter/son have a low birth weight?	☐ Yes, low birth weight ☐ No
How much did your daughter/son weigh at birth?	____________________

Here is a list of common pregnancy complications. I ask you to mark all that apply to the pregnancy of your daughter/son [name] and indicate approximately in which trimester the event occurred:

Complication	Did occur?	Trimester		
High Blood Pressure	☐ Yes	☐ First	☐ Second	☐ Third
Gestational Diabetes	☐ Yes	☐ First	☐ Second	☐ Third
Infections (which?_______________)	☐ Yes	☐ First	☐ Second	☐ Third
Preeclampsia	☐ Yes	☐ First	☐ Second	☐ Third
Preterm Labor	☐ Yes	☐ First	☐ Second	☐ Third
Depression & Anxiety	☐ Yes	☐ First	☐ Second	☐ Third
Severe, persistent nausea and vomiting	☐ Yes	☐ First	☐ Second	☐ Third
Severe anemia	☐ Yes	☐ First	☐ Second	☐ Third

Additional events that might have influenced birthweight

	Did occur?	Trimester		
Alcohol use during pregnancy	☐ Yes	☐ First	☐ Second	☐ Third
Smoking during pregnancy	☐ Yes	☐ First	☐ Second	☐ Third
Excess caffeine (>3 per day)	☐ Yes	☐ First	☐ Second	☐ Third
Any traumatic event (risk for life)	☐ Yes	☐ First	☐ Second	☐ Third
Any stressful event	☐ Yes	☐ First	☐ Second	☐ Third

Here is a list of common labor complications. I ask you to mark all that apply to the birth of your daughter/son [name]

Complication	Did occur?	Was it severe?
Preterm labor (before 37th week)	☐ Yes	☐ Yes
Postterm (after the 42nd week)	☐ Yes	☐ Yes
Abnormal presentation	☐ Yes	☐ Yes
Intra-amniotic infection	☐ Yes	☐ Yes
Massive bleeding	☐ Yes	☐ Yes
Convulsions during labor	☐ Yes	☐ Yes
Prolonged labor (>12 hours)	☐ Yes	☐ Yes
Placenta previa	☐ Yes	☐ Yes
Very high fever	☐ Yes	☐ Yes
Fetal distress	☐ Yes	☐ Yes
Retained placenta	☐ Yes	☐ Yes
Post partum hemorrhage	☐ Yes	☐ Yes
Labor started with medication (induction of labor)	☐ Yes	☐ Yes
Use of forceps or vacuum device	☐ Yes	☐ Yes
Caesarean section	☐ Yes	☐ Yes

Additional information

Gestational age at birth _________________

Weight at birth _________________

Did your daughter/son have any problems within the first 12 months?

Here is a list of common problems within the first 12 months. I ask you to mark all that apply to your daughter/son [name]

Problem	Did occur?	Was it severe?	Have they required hospital admission?
Frequent colds, coughs, fevers	☐ Yes	☐ Yes	☐ Yes Age ________
Frequent vomiting	☐ Yes	☐ Yes	☐ Yes Age ________
Delay in walking	☐ Yes	☐ Yes	☐ Yes Age ________
Delay in speech	☐ Yes	☐ Yes	☐ Yes Age ________
Temper tantrum crisis	☐ Yes	☐ Yes	☐ Yes Age ________
Frequent utterly crying or inconsolable tears	☐ Yes	☐ Yes	☐ Yes Age ________
Seizures	☐ Yes	☐ Yes	☐ Yes Age ________
Picky eating	☐ Yes	☐ Yes	☐ Yes Age ________
Baby food refusal	☐ Yes	☐ Yes	☐ Yes Age ________
Difficulties of breastfeeding	☐ Yes	☐ Yes	☐ Yes Age ________
PICA (eating non-food items)	☐ Yes	☐ Yes	☐ Yes Age ________

Did your daughter/son have any health issues from 1 to 6 years of age?

Here is a list of common health issues from 1 to 6 years of age. I ask you to mark all that apply to your daughter/son [name]

Problem	Did occur?	Was it severe?	Have they required hospital admission?
S/he was diagnosed with autism	☐ Yes	☐ Yes	☐ Yes Age ________
S/he was diagnosed with attention deficit hyperactivity disorder	☐ Yes	☐ Yes	☐ Yes Age ________
S/he was diagnosed with avoidant/restrictive food intake disorder (ARFID), a feeding disorder in which people avoid eating certain foods, or restrict their diets to the point it ultimately results in nutritional deficiencies.	☐ Yes	☐ Yes	☐ Yes Age ________
S/he was diagnosed with celiac disease	☐ Yes	☐ Yes	☐ Yes Age ________
S/he had any food-related allergies	☐ Yes	☐ Yes	☐ Yes Age ________
S/he was diagnosed with infantile obesity	☐ Yes	☐ Yes	☐ Yes Age ________
S/he suffered from prolonged or acute infections	☐ Yes	☐ Yes	☐ Yes Age ________
S/he was diagnosed with epilepsy	☐ Yes	☐ Yes	☐ Yes Age ________
S/he was diagnosed with asthma	☐ Yes	☐ Yes	☐ Yes Age ________
S/he suffered from recurrent gastroenteritis	☐ Yes	☐ Yes	☐ Yes Age ________

Did your daughter/son have any health issues from 7 to 15 years of age?

Here is a list of common health issues from 7 to 15 years of age. I ask you to mark all that apply to your daughter/son [name]

Problem	Did occur?	Was it severe?	Have they required specialized medical assessment?
S/he had school difficulties	☐ Yes	☐ Yes	☐ Yes Age _______
S/he was diagnosed with learning disabilities	☐ Yes	☐ Yes	☐ Yes Age _______
S/he was diagnosed with obsessive-compulsive disorder	☐ Yes	☐ Yes	☐ Yes Age _______
S/he was a victim of bullying	☐ Yes	☐ Yes	☐ Yes Age _______
S/he engaged in acts of violence or was frequently reported for disruptive behavior	☐ Yes	☐ Yes	☐ Yes Age _______
S/he showed self-injury behavior or self-harm	☐ Yes	☐ Yes	☐ Yes Age _______
S/he menaced or attempted suicide	☐ Yes	☐ Yes	☐ Yes Age _______
S/he showed a habit of using alcohol	☐ Yes	☐ Yes	☐ Yes Age _______
S/he started smoking	☐ Yes	☐ Yes	☐ Yes Age _______
S/he showed a habit of using illicit drugs (i.e., hashish, marijuana)	☐ Yes	☐ Yes	☐ Yes Age _______
S/he was diagnosed with teenage obesity	☐ Yes	☐ Yes	☐ Yes Age _______
S/he showed intense, recurrent, or acute anxiety	☐ Yes	☐ Yes	☐ Yes Age _______
S/he showed intense or recurrent mood swing	☐ Yes	☐ Yes	☐ Yes Age _______
S/he suffered from insomnia or sleep disorders	☐ Yes	☐ Yes	☐ Yes Age _______
S/he suffered from depression or melancholia	☐ Yes	☐ Yes	☐ Yes Age _______
S/he suffered from an abrupt psychotic breakdown	☐ Yes	☐ Yes	☐ Yes Age _______
S/he sudden withdrawal from usual extra-curricular activities (sports, hobbies, outbound with friends)	☐ Yes	☐ Yes	☐ Yes Age _______
S/he started a rigid diet	☐ Yes	☐ Yes	☐ Yes Age _______
S/he started to avoid public lunch or dinner	☐ Yes	☐ Yes	☐ Yes Age _______
S/he started to complain of poor shape or showing body dissatisfaction	☐ Yes	☐ Yes	☐ Yes Age _______
S/he negotiated tattoos and body piercings without parental approval	☐ Yes	☐ Yes	☐ Yes Age _______
S/he ran away from home	☐ Yes	☐ Yes	☐ Yes Age _______
S/he skipped school without notifying the parents	☐ Yes	☐ Yes	☐ Yes Age _______
S/he was reported for truancy	☐ Yes	☐ Yes	☐ Yes Age _______
S/he suffered an unexpected mourning, whether related to a relative or a pet	☐ Yes	☐ Yes	☐ Yes Age _______
S/he repeatedly moved school	☐ Yes	☐ Yes	☐ Yes Age _______
S/he moved house often	☐ Yes	☐ Yes	☐ Yes Age _______

Did a sister or a brother of your daughter/son have any health issues?

Here is a list of common health issues. I ask you to mark all that apply to.

Problem	Did occur?	Was it severe?	Have they required hospital admission?
S/he was diagnosed with autism	☐ Yes	☐ Yes	☐ Yes Age _______
S/he was diagnosed with attention deficit hyperactivity disorder	☐ Yes	☐ Yes	☐ Yes Age _______
S/he was diagnosed with celiac disease	☐ Yes	☐ Yes	☐ Yes Age _______
S/he had any food-related allergies	☐ Yes	☐ Yes	☐ Yes Age _______
S/he was diagnosed with infantile obesity	☐ Yes	☐ Yes	☐ Yes Age _______
S/he had school difficulties	☐ Yes	☐ Yes	☐ Yes Age _______
S/he was diagnosed with learning disabilities	☐ Yes	☐ Yes	☐ Yes Age _______
S/he was diagnosed with obsessive-compulsive disorder	☐ Yes	☐ Yes	☐ Yes Age _______
S/he was a victim of bullying	☐ Yes	☐ Yes	☐ Yes Age _______
S/he engaged in acts of violence or was frequently reported for disruptive behavior	☐ Yes	☐ Yes	☐ Yes Age _______
S/he showed self-injury behavior or self-harm	☐ Yes	☐ Yes	☐ Yes Age _______
S/he menaced or attempted suicide	☐ Yes	☐ Yes	☐ Yes Age _______
S/he showed a habit of using alcohol	☐ Yes	☐ Yes	☐ Yes Age _______
S/he started smoking	☐ Yes	☐ Yes	☐ Yes Age _______
S/he showed a habit of using illicit drugs (i.e., hashish, marijuana)	☐ Yes	☐ Yes	☐ Yes Age _______
S/he was diagnosed with teenage obesity	☐ Yes	☐ Yes	☐ Yes Age _______
S/he showed intense, recurrent, or acute anxiety	☐ Yes	☐ Yes	☐ Yes Age _______
S/he showed intense or recurrent mood swing	☐ Yes	☐ Yes	☐ Yes Age _______
S/he suffered from insomnia or sleep disorders	☐ Yes	☐ Yes	☐ Yes Age _______
S/he suffered from depression or melancholia	☐ Yes	☐ Yes	☐ Yes Age _______
S/he suffered from an abrupt psychotic breakdown	☐ Yes	☐ Yes	☐ Yes Age _______
S/he received a diagnosis of eating disorder	☐ Yes	☐ Yes	☐ Yes Age _______
Which one?	____		

Was your daughter/son ever diagnosed with an eating disorder?	☐ Yes Age _______
Which one?	_____________________
Did you ever notice abnormal eating habits of your daughter/son, i.e., excessive food weighing, hoarding food, pushing food around in plates to postpone eating?	☐ Yes Age _______
Did you ever notice self-induced vomiting, laxative or diuretic abuse by your daughter/son?	☐ Yes Age _______
Did you ever notice intense body dissatisfaction in your daughter/son?	☐ Yes Age _______
Did you ever notice intense or recurring weight or shape preoccupation in your daughter/son?	☐ Yes Age _______
